# Fatigue

**Published:** 1896-10

**Authors:** 


### Fatigue.

The experiments of Masse, of Turin, and of Michael Foster, the physiologist, have shown that the sense of fatigue is due to poisoning of the cerebrum by the products of retrograde metamorphosis. "The blood of a tired animal is poisoned, and when injected into another animal causes the phenomena of fatigue." The toxicity of the blood may become so great as to prove fatal, as was shown by Foster in rabbits that had been hunted to death.—*Dr. Bartour, in American Practitioner and News.*

